# Endovascular Treatment of an Aortic Traumatic Double Rupture

**DOI:** 10.15171/jcvtr.2015.08

**Published:** 2015-03-29

**Authors:** Domenico Attinà, Francesco Buia, Vincenzo Russo, Emanuele Pilato, Luigi Lovato, Roberto Di Bartolomeo, Maurizio Zompatori

**Affiliations:** ^1^ Cardio-Thoracic-Vascular Department, Cardiothoracic Radiology Unit, University Hospital S.Orsola-Malpighi, Bologna, Italy; ^2^ Cardio-Thoracic-Vascular Department, Cardiac Surgery Unit, University Hospital S.Orsola-Malpighi, Bologna, Italy

**Keywords:** Trauma, Thoracic Aorta, Aortic Rupture, Endovascular Stent Graft, Endovascular Procedure, Emergency

## Abstract

Traumatic thoracic aortic rupture is a life-threatening condition; aortic isthmus is the most common site of rupture, but in rare cases traumatic injury can localize elsewhere, such as at aortic arch or at the level of the diaphragm. In the past few years, endovascular treatment of traumatic aortic injury became a safe procedure, with lower mortality and complication, if compared with open surgery. We report a case of a 40-year-old-man admitted to emergency department after a violent car crash in which an aortic traumatic double rupture was successfully treated with two endovascular stent-grafts coverage.

## Case report

A 40 years old man was admitted to emergency department after a violent car crash. The patient has been subjected to multiple injuries: abdominal wall contusion; pelvic, low extremities and rib fractures. Moreover he was also hemodynamically unstable: he presented tachycardia (heart rate 120 bpm) and hypotension (blood pressure 90/60 mmHg). Arterial blood gas analysis showed: pH 7,50; pCO2 29 mmHg; pO2 64 mmHg; Hb 9,4 g/dL; SaO2 95%; Hct 28%. Serum creatinine was 0,5 mg/dL.

The initial ultrasound evaluation excluded any abdominal solid organ injuries and a left pleural effusion was detected. A CT scan of the thorax showed a post-traumatic complete rupture of the aortic isthmus, close to the left subclavian artery origin (distance: 18 mm), along with hemomediastinum and hemorrhagic left pleural effusion. Due to the presence of breathing and motion artifacts, a further injury of the aorta could not be excluded.

**Figure 1:**
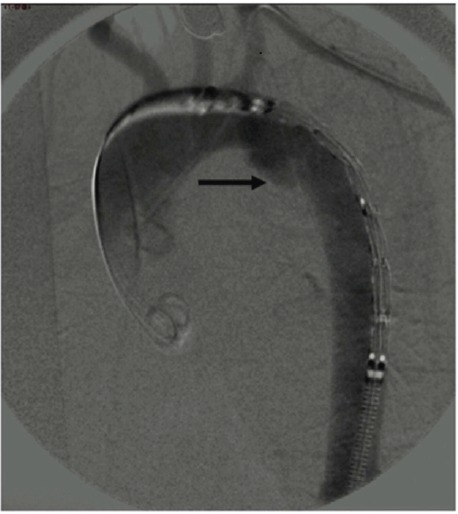
The angiography performed before stent-graft placement confirms the aortic isthmus rupture, close to left subclavian artery (arrow). The stent-graft is still contained inside the sheath.

The patient was transfused with 2 units of red blood cells and immediately transferred in operating room for thoracic endovascular aortic repair. Under general anaesthesia, with tracheal intubation and mechanical ventilation, the right common femoral artery was surgically approached to serve as access route. A 260-cm-long 0.035-inch super stiff guide wire was used to insert a stent-graft, contained inside a sheath, in the thoracic descending aorta (Valiant Thoracic Stent Graft with the Captivia Delivery System, Medtronic Vascular, Inc. Santa Rosa, CA: VAMF2626C100TE). The stent-graft size (26 mm × 100 mm) was determined on the basis of the CT scan and was oversized by 15% in relation to the diameter of the native aorta, to ensure a satisfactory seal. At the same time, the left brachial artery was catheterized with a 0.035-inch hydrophile guide wire and an angiography catheter was placed in the ascending aorta. The angiography of the whole thoracic aorta, taken from a left anterior oblique view, confirmed the presence of aortic isthmus rupture (Figure 1).

Transesophageal echocardiography, which could be used to guide the procedure, to reduce the amount of injected contrast and to rule out other aortic injuries, was not available in that emergency setting. Therefore, the stent graft was implanted inside the thoracic aorta, close to the origin of the left subclavian artery, under fluoroscopic monitoring and with mean arterial pressure less than 70 mmHg.

**Figure 2:**
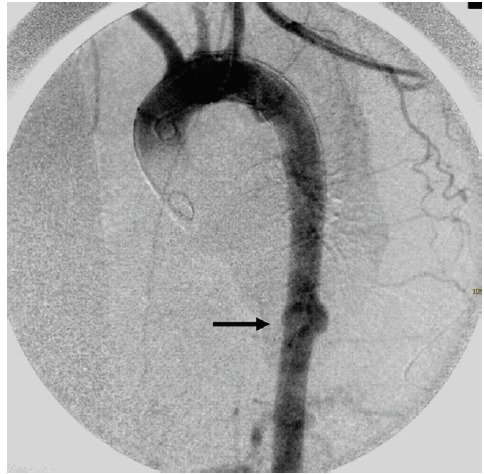
The angiography performed with a larger field of view, after the placement of the first stent-graft, highlights a simultaneous aortic injury at the level of the diaphragm (arrow).

During the post-procedure angiography, performed with a larger field of view, a simultaneous aortic injury was noted at the level of the diaphragm (Figure 2). Also the second lesion was treated with deployment of a second tapered stent-graft (Valiant Captivia Medtronic VAMC3026C150TE), overlapping the distal 5 cm of the first prosthesis. The second stent-graft size (proximal size 30 mm, distal size 26 mm, length 150 mm) was chosen to obtain an optimal sealing with the previously positioned prosthesis.

**Figure 3:**
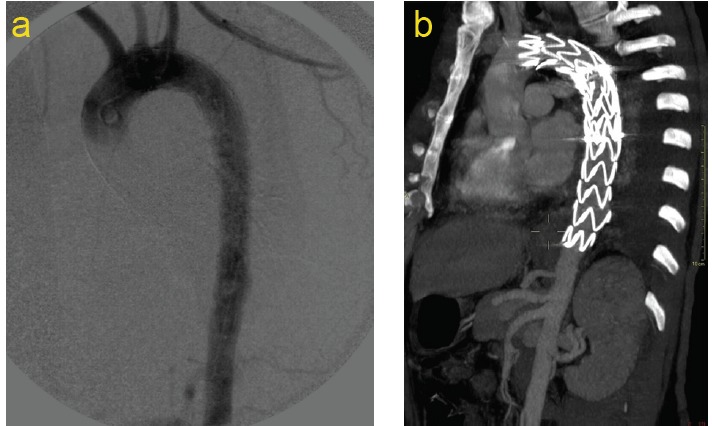
(a) The angiography performed after the placement of the second stent-graft shows the complete coverage of both the pseudo-aneurysms. (b) Post-operative CT angiography performed at 7 days after endovascular treatment further confirms fully deployment of both the prostheses and the absence of endoleaks.

No signs of endoleak were detected by the second post-procedural angiography (Figure 3a) and by CT scan performed after a week (Figure 3b).

After the endovascular procedure, given the presence of a significant hemothorax, a left pleural drainage was placed.

The patient was hospitalized for 40 days, during which underwent orthopaedic treatment of femur and pelvic fractures and rehabilitation. After he was discharged in good condition.

Written patient’s informed consent was obtained for publication of this report.

## Discussion

Traumatic thoracic aortic rupture is a life-threatening condition; aortic isthmus rupture is the most common thoracic aorta emergency in males in the fourth decade and, after head injuries, is the most common cause of death in traumatic accidents.^1^

In recent years, car and motorcycle crashes have accounted for more than 75% of traumatic aortic rupture, but there are also other causes like fall from height, direct blow, explosion and compression by a heavy object.^2,3^

Thoracic aortic rupture can be complete or incomplete. In the first case, death is often instantaneous; when the rupture is incomplete, the integrity of adventitia and periadventitial tissue can evolve into a post-traumatic pseudoaneurysm. Aortic injury is the consequence of traumatic rapid deceleration and is commonly localized in the aortic isthmus; in this region the mobile tract of thoracic aorta joins the fixed arch and the insertion of the ligamentum arteriosus.^3^

Aortic rupture is less commonly localized in aortic root and in this case can be associated with aortic valve injury, coronary tears and cardiac contusion or rupture.^4^

In nearly 2% of cases, aortic traumatic injury is localized at the level of the diaphragm^5,6^; simultaneous lesions are very rare, with only one other case (to our knowledge) previously reported in medical literature.^7^

In the past, aortic traumatic injury was considered a surgical emergency hampered by high operative mortality. In the last few years endovascular treatment of traumatic aortic injury became a sure option for those patients, with lower mortality and complication if compared with open surgery, without necessity of thoracotomy and aortic clamping and, consequentially with less risk of medullary ischemia and worsening of other injuries.^8^

Cerebrospinal fluid drainage could be used to avoid the risk of neurologic complications in cases with whole descending aorta stenting, but its use is still impractical in life-threatening conditions that require emergency treatment, because its placement could delay further the lifesaving procedure.

Moreover, endovascular repair of aortic traumatic lesion is a feasible and relatively safe technique, less invasive than surgery and with acceptable medium-term results^9^; long-term results are necessary to definitively assess reliability of stent-graft materials and improvement in patient survival.

## Conclusion

Traumatic aortic injuries are medical and surgical emergencies, which benefit from endovascular treatment. A complete and accurate assessment of the aorta is indicated to perform a safe and successful procedure, but it is not always possible because of patient’s critical condition. In our case, an unrecognized diaphragmatic aortic lesion was highlighted during the post-procedure angiography, performed with a larger field of view and a second stent-graft was successfully implanted.

We therefore recommend in all patient with traumatic aortic rupture to perform a post-procedural angiography of the entire thoraco-abdominal aorta to highlight any vascular simultaneous lesions, previously undetected.

## Ethical issues

The study was approval by the Local Ethics Committee.

## Competing interests

Authors declare no conflict of interests in this study.

## References

[1] Bertrand S, Cuny S, Petit P, Trosseille X, Page Y, Guillemot H (2008). Traumatic rupture of thoracic aorta in real-world motor vehicle crashes. Traffic Inj Prev.

[2] Botta L, Russo V, Savini C, Buttazzi K, Pacini D, Lovato L (2008). Endovascular treatment for acute traumatic transection of the descending aorta: focus on operative timing and left subclavian artery management. J Thoracic Cardiovasc Surg.

[3] Fattori R, Napoli G, Lovato L, Russo V, Pacini D, Pierangeli A (2002). Indication for, timing of, and results of catheter-based treatment of traumatic injury to the aorta. AJR.

[4] Alkadhi H, Wildermuth S, Desbiolles D, Schertler T, Crook D, Marincecek B (2004). Vascular emergencies of the thorax after blunt and iatrogenic trauma: multi-detector row CT and three-dimensional imaging. Radiographics.

[5] Wintermark M, Wicky S, Schnyder P (2002). Imaging of acute traumatic injuries of the thoracic aorta. Eur Radiol.

[6] Ben-Menachem Y (1993). Rupture of the thoracic aorta by broadside impacts in road trafﬁc and other collisions: further angiographic observations and preliminary autopsy ﬁndings. J Trauma.

[7] Daghfous A, Daiki M, Ben Khélifa El Moncer R, Maarouf M, Felah S, Zoghlami A (2014). Acute traumatic thoracic aortic rupture in double localisation. Ann Cardiol Angeiol.

[8] Orend KH, Pamler R, Kapfer X, Liewald F, Gorich J, Sunder-Plassmann L (2002). Endovascular Repair of Traumatic Descending Aortic Transection. J Endovasc Ther.

[9] Dake MD, Miller DC, Mitchell RS, Semba CP, Moore KA, Sakai T (1998). The “first generation” of endovascular stent-grafts for patients with aneurysms of the descending thoracic aorta. J Thorac Cardiovasc Surg.

